# Providing value to patients and providers via a pediatric statewide antibiogram in South Carolina

**DOI:** 10.1017/ash.2023.149

**Published:** 2023-04-24

**Authors:** Pamela Bailey, Kayla Antosz, Robert Daniels, Andrew B. Gainey, Anna Kathryn Burch

**Affiliations:** 1 Prisma Health Midlands, Columbia, South Carolina; 2 University of South Carolina School of Medicine, Columbia, South Carolina; 3 Antimicrobial Stewardship Collaborative of South Carolina (ASC-SC), Columbia, South Carolina; 4 Medical University of South Carolina Columbia, Columbia, South Carolina; 5 University of South Carolina College of Pharmacy, Columbia, South Carolina; 6 Prisma Health Children’s Hospital–Midlands, Columbia, South Carolina; 7 South Carolina Department of Health and Environmental Control (DHEC), Columbia, South Carolina

## Abstract

**Objective:**

Antimicrobial stewardship has special challenges in particular populations and facilities, including pediatrics. We sought to augment the information available to antimicrobial stewardship programs (ASPs) by created a cumulative statewide antibiogram for neonatal and pediatric populations.

**Methods:**

In the Antimicrobial Stewardship Collaborative of South Carolina (ASC-SC), we created statewide antibiograms, including a separate antibiogram accounting for the pediatric and neonatal intensive care unit (NICU) populations. We collated data from the 4 pediatric and 3 NICU facilities in the state to provide a cumulative statewide antibiogram.

**Results:**

Methicillin-susceptible Staphylococcus aureus was more prevalent than methicillin-resistant Staphylococcus aureus. Pseudomonas aeruginosa, Citrobacter koserii, and Acinetobacter baumannii were isolated in only 1 NICU.

**Conclusions:**

These antibiograms should improve empiric prescribing in both the inpatient and outpatient setting, providing data in some areas that historically do not have pediatric antibiogram to inform prescribing. The antibiogram alone is not sufficient independently to improve prescribing but is one important aspect of stewardship in the pediatric population of South Carolina.

Antimicrobial resistance is a well-documented threat to public health worldwide, with ∼3 million deaths annually in the United States related to antimicrobial-resistant infections and ∼$4.6 billion spent in healthcare costs.^1^ The Centers for Disease Control and Prevention notes issues with antimicrobial resistance data, in terms of tracking antimicrobial resistance, appropriately collecting antimicrobial use, and publishing performance data about appropriate use.^1^ A part of the *National Action Plan for Combating Antibiotic Resistant Bacteria* ^2^ includes expanding surveillance data and supporting research to improve the responsible use of antibiotics across settings (both healthcare and community) and to translate important findings into practice.

There are special challenges in implementing antimicrobial stewardship programs across differing healthcare facilities include limitations in staff, infrastructure, and resources.^3^ In South Carolina, the Antimicrobial Stewardship Collaborative (ASC-SC), a collaboration of infectious diseases (ID) physicians (pediatric and adult) and pharmacists, who leverage specialized ID resources for facilities that do not have such support, particularly in rural areas of the state.

One of the initiatives that ASC-SC undertakes yearly is the creation of statewide and regional antibiograms. The antibiogram is an essential resource to track changes in antimicrobial resistance and to guide empiric antimicrobial therapy.^4^ While most acute-care hospitals provide antibiograms for inpatients, there is a dearth of data outside those institutions. Localized or regional antibiograms offer significant value to communities and prescribers as patients frequently transfer between facilities within regions.^5,6^ However, these cumulative antibiograms have limitations, and generally cannot meet the formal antibiogram standards set by the Clinical and Laboratory Standards Institute (CLSI).^7^ These limitations are significant barriers for small pediatric hospitals, outpatient clinics, and long-term care facilities, which frequently lack the number of isolates in addition to simply lacking culture data for patients.^7–9^ Regardless of these limitations, cumulative antibiograms have previously been validated to have meaningful results for communities.^8,10,11^ They are a practical and inexpensive tool to aid clinicians in empiric antimicrobial choices, and with significant clinical significance.^5,6,10^

In pediatrics, cumulative antibiograms have previously been used and are a useful tool for institutions that do not otherwise have the ability to generate a CLSI standardized antibiogram.^12,13^ The purpose of this project was to generate a statewide pediatric and NICU antibiogram to aid in empiric antimicrobial prescribing and identify resistance patterns across the state.

## Methods

ASC-SC collected both pediatric and neonatal ICU (NICU) antibiograms from all 4 children’s hospitals in the state (3 NICU specific antibiograms, 4 pediatric antibiograms) for 2020. The aggregate information was provided from the hospital laboratory, and therefore includes predominantly emergency department and inpatient specimens; few outpatient clinics utilize the hospital’s laboratory services. South Carolina has 5 perinatal referral centers (high-acuity NICUs, level III or IV). For pediatric patients in South Carolina, these are the 4 major referral children’s hospitals in South Carolina (level I or II trauma centers); 3 of these are academic medical centers that serve as cystic fibrosis referral centers and serve a significant proportion of oncology and immunocompromised patients. One hospital’s antibiogram had a few isolates that contained both 2019 and 2020 data, and both years were included in the antibiogram. Each antibiogram was deconstructed into individual isolates and then combined into a single statewide antibiogram. All isolates were included regardless of the number of isolates reported in each facility’s antibiogram. The 3 NICUs had 101, 48, and 82 beds each. The pediatric hospitals had ∼700 beds in total (170, 100, 180, and 250 beds at individual institutions). Only 1 of the 4 institutions has dedicated pediatric antimicrobial stewardship physicians or pharmacists.

## Results

**Fig. 1.**
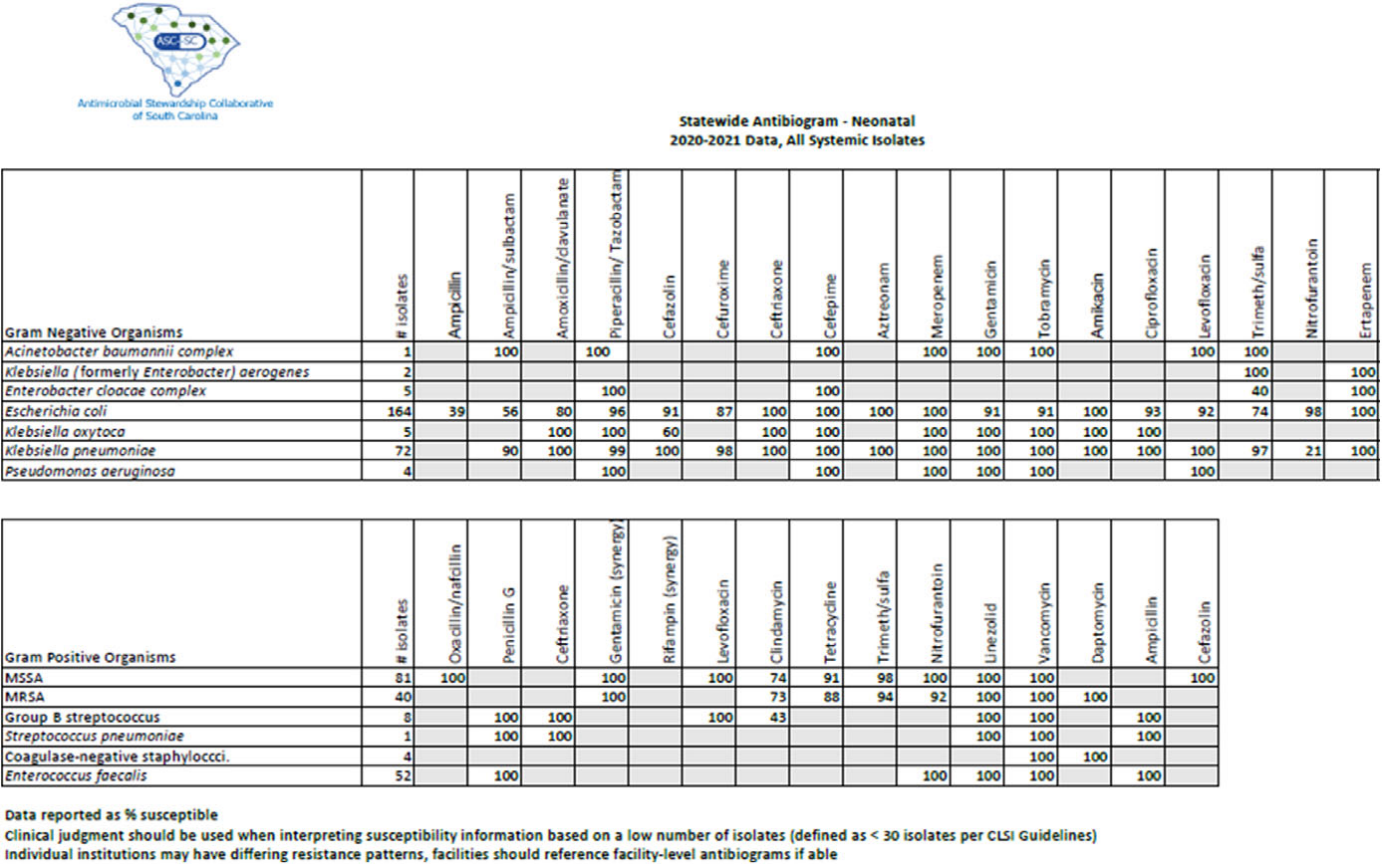
Statewide neonatal antibiogram, 2020–2021.

**Fig. 2.**
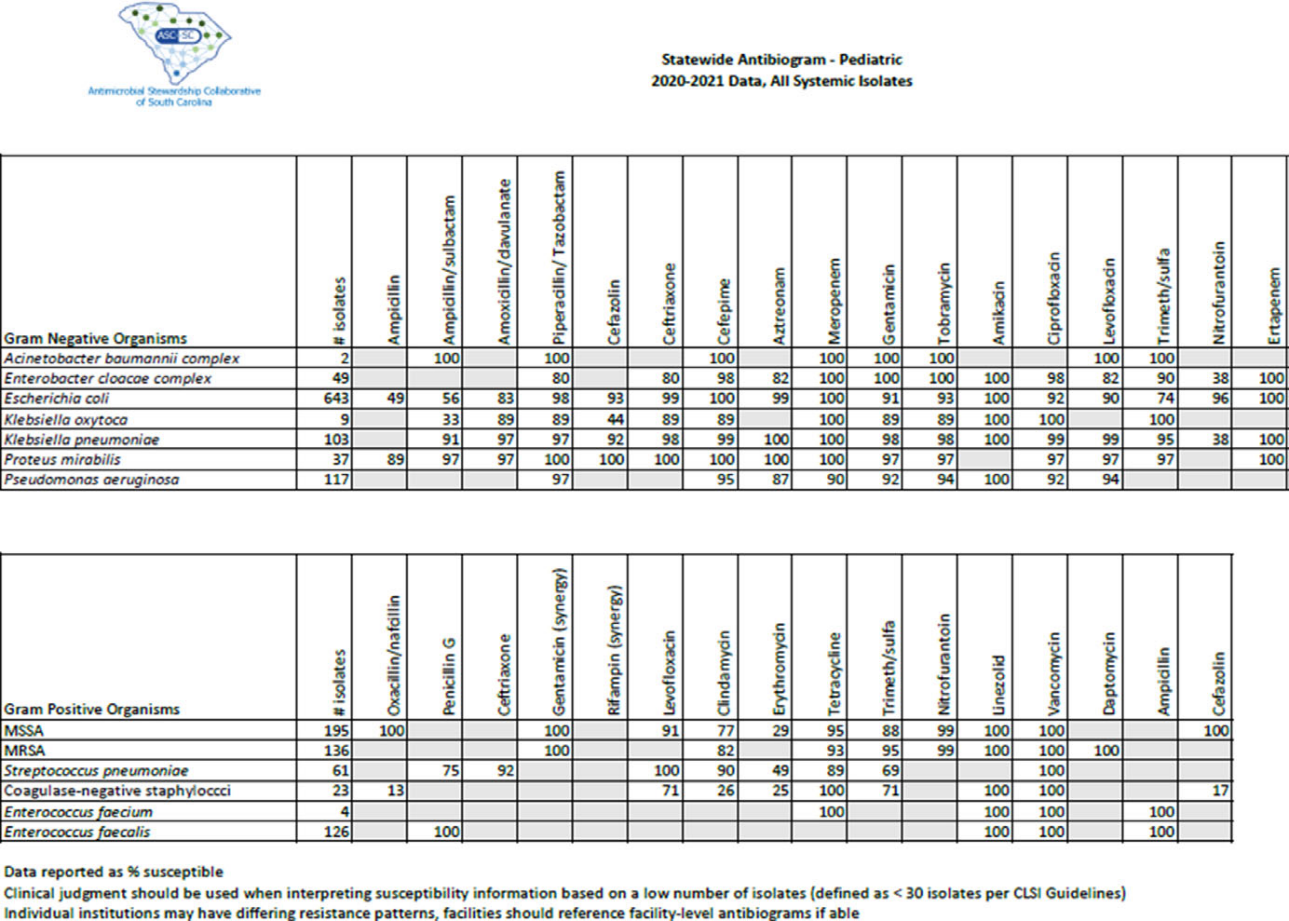
Statewide pediatric antibiogram, 2020–2021.

Overall, *Escherichia coli* was the most frequently reported organism, with 164 isolates in the NICU antibiogram and 643 isolates in the pediatric antibiogram. Methicillin-susceptible *Staphylococcus aureus* (MSSA) was more prevalent than methicillin-resistant *Staphylococcus aureus* (MRSA) in both the neonatal and pediatric populations, with 81 and 195 isolates, respectively. *Pseudomonas aeruginosa*, *Citrobacter koseri*, and *Acinetobacter baumannii* were isolated in only 1 NICU. No extended-spectrum β-lactamase infections nor carbapenem-resistant infections were reported. Further information is shown in the NICU and pediatric antibiograms in Figures 1 and 2, respectively. Notably, only 2 of the 4 institutions reported coagulase-negative *Staphylococcus* results.

## Discussion

This initiative is an attempt to improve empiric antimicrobial prescribing for frontline pediatricians prescribing the bulk of antibiotics in the United States.^14^ Provision of antibiograms to community pediatricians is an important antibiotic stewardship strategy, particularly because outpatient antibiotic prescribing is highly variable and/or is often inappropriate and because access to an antibiogram is perceived as valuable for identifying antimicrobial resistance and guiding empiric antibiotic selection.^15^ Education will accompany the antibiogram to optimize antimicrobial prescribing. We have distributed the antibiograms via our website and by email to appropriate providers, and we have provided information sessions about antibiograms through the ASC-SC webinar series.

Evidence should drive the practice of medicine, yet practitioners frequently do not have the evidence they need to provide the best care. A cross-sectional survey of 85 pediatric medical residents from 2 pediatric hospitals showed that only 57% of the residents had ever utilized an antibiogram.^16^ Similarly, a second survey of pediatric medical residents at a single pediatric hospital identified that 50.9% of residents had never utilized an antibiogram.^17^ In general practice, a survey of Illinois pediatricians demonstrated that only 25% had access to a pediatric-specific antibiogram, and this access was associated with access to subspecialty training and Chicago-based practitioners.^15^ In the same study, 40% of respondents indicated that they did not feel well informed about national or local antibiotic resistance rates for pathogens causing common pediatrics infections.^15^

In terms of antimicrobial resistance and organisms that may be more clinically concerning, a few isolates (*Pseudomonas aeruginosa, Citrobacter koserii*, and *Acinetobacter baumannii*) were isolated in only 1 NICU. Furthermore, previous literature noted a national trend in pediatric *Staphylococcus aureus* to be 50% MSSA and 50% MRSA.^13^ However, this statewide antibiogram shows MSSA with a higher prevalence rate than MRSA. These data may be important in regard to neonatal transfers; no other NICUs in the state have isolated such organisms. Furthermore, NICU data can be strongly patient specific via hospital-acquired mechanisms, which may limit its applicability statewide. A footnote on the antibiogram directs users to preferentially utilize the institution’s antibiogram should it be available to the prescriber because that information is likely more specific for their individual patient. In the educational initiatives, we emphasize that the antibiogram provides important information to consider when prescribing empiric antimicrobials, but clinical judgment in individual situations is critical.

The limitations of antibiograms include that they can be heavily influenced by a chronically ill population, especially if the number of isolates is small. This factor significantly affects use. Some pediatricians indicated that they would not use antibiograms due to generalizability to their population because the antibiograms represent hospitalized patients, frequently reflect adults only, or are poorly representative of their geographic area.^15^ Traditional (ie, inpatient hospital) antibiograms may also miss differences in the syndromes under treatment (ie, respiratory vs blood vs urine), special populations like pediatrics or chronically hospitalized patients, or treatment setting (ie, inpatient vs outpatient).^9,15,^ ^18^ Some pediatric antibiograms have been built that separate healthy children from more medically complex children, and pediatricians strongly favor antibiograms that separate medically complex patient from healthy populations.^9,19^ Research into syndromic antibiograms (ie, bloodstream, respiratory infections) to further personalize empiric antibiotic prescribing guided by the antibiogram is ongoing.^9^

Although constructing a statewide antibiogram did increase some numbers of isolates, further understanding of their applicability in clinical practice is warranted. A division remains between outpatient and inpatient representation in the data in the antibiogram. Also, antibiograms should not be the sole driving force for selecting empiric therapy; a patient’s individual microbiologic history can provide a greater insight. Other factors to consider outside of using an antibiogram include pediatric pharmacodynamic and pharmacokinetic data and pediatric safety data.

Additionally, guideline adherence and a better understanding of outpatient antimicrobial prescribing practices is critical to appropriate antimicrobial prescribing. A better understanding of these aspects will help drive antimicrobial stewardship initiatives and quality improvement projects like ASC-SC. Additionally, there are significant racial disparities in antimicrobial prescribing in pediatrics that need to be further explored.^20^ The COVID-19 pandemic has disrupted significant strides made in inpatient stewardship; however, outpatient prescriptions have decreased which may have interesting implications.^21^

The limitations of this project include potentially different methodologies used to collect and report each institution’s antibiogram, as noted in the CLSI standards warning against cumulative antibiograms.^7^ Some facilities’ antibiograms had <30 isolates (most frequently seen in the NICU), and these were all included to provide numerical significance. Additionally, we collected completed antibiograms from facilities, which may have been modified to exclude information at the facility level that was not consistent across all 4 facilities.

In conclusion, statewide pediatric antibiograms can be a helpful tool to improve antimicrobial prescribing and monitor antimicrobial resistance trends throughout the state. In addition to providing a statewide antibiogram, education will be required to maximize appropriate antimicrobial prescribing in this population.
